# The Economic Cost of Nursing Care of Palliative Patients in the Emergency Department

**DOI:** 10.3390/healthcare13040421

**Published:** 2025-02-15

**Authors:** Tânia dos Santos Afonso, Manuel Luís Capelas, Lurdes Martins

**Affiliations:** Center for Interdisciplinary Research in Health (CIIS), Faculty of Health Sciences and Nursing, Universidade Católica Portuguesa, 1649-023 Lisbon, Portugal; luis.capelas@ucp.pt (M.L.C.); lurdes.martins@ucp.pt (L.M.)

**Keywords:** palliative care, emergency department, cost, nursing care

## Abstract

(1) Background: The economic cost of nursing care to palliative patients in avoidable hospital admission is unknown. (2) Methods: An observational, retrospective, and analytical study was used encompassing the economic cost of nursing care in a general hospital emergency department that uses descriptive and inferential statistical analysis. (3) Results: We have a sample of 273 palliative patients in preventable hospital admissions (76.3%, CI 95%: 71.7–80.8), deceased, in 2019. With a median of 84 years, about 80% were admitted home after 5 h of complaints, mainly due to respiratory symptoms. Patients remained for approximately 24 h in the emergency department, totaling a median of EUR 180.98 in nursing care costs per admission. (4) Conclusions: It was evident that with the knowledge obtained about palliative economic nursing costs, we could increase results, manage resources, and consolidate PC integration in health systems, as this study proved. This study was not registered.

## 1. Introduction

Palliative care is an integral part of health systems, defended by evidence that it best responds to an increasingly large group of patients [1].

Population aging and increasing morbidity support the need for this type of specialized care, emphasizing the importance of strengthening palliative care [2]. Palliative care is based on a care philosophy and intervention with added value in the quality and efficiency of health systems, reiterating that they do not increase costs in this area [3,4,5,6,7,8].

Transitions to the hospital space have been studied despite the evident need for more data at a national level. There is consensus in the literature that the weak development of community support contributes not only to an increase in hospital admissions but also to an increase in healthcare costs [9,10,11,12].

The relevance of defining the economic cost associated with nursing care for palliative patients admitted to the emergency department in admissions considered avoidable is based on existing knowledge about these transitions. The increasing transition of death and palliative emergencies to the hospital space is reinforced [13,14,15], with data relating to the costs associated with nursing care for palliative patients, in hospital general urgency, in Portugal, being unknown when these emergencies are avoidable.

The knowledge explored regarding the aspects inherent to the admission of palliative patients in a general hospital emergency department, focusing on the economic costs associated with nursing care provided during an avoidable emergency, is innovative and based on the little knowledge regarding this context in Portugal.

Not only does the lack of this knowledge justify the path taken, but other arguments can also be stated: lack of knowledge of the personal, social, and economic impact of avoidable hospital emergency admissions; the increasing transition of death and emergencies of palliative patients to the hospital space, with data on the costs associated with nursing care for palliative patients being non-existent; the importance of specifying the impact of increasing resources in palliative care on health outcomes and the importance of specifying the economic impact on cost reduction, achieved through investment in teams specialized in palliative care.

The knowledge explored will support better coordination between institutions and contexts and enhance the quality of care at the end of life. These data compete with the notion that the accessibility of palliative care resources directly influences patients’ quality of life and comfort.

Recognizing the economic pressure on the influence of the management of hospital institutions, reducing the use of hospital space is one of the objectives of health decision-makers. Therefore, integrating palliative care at different levels of the health system is fundamental. These data can be combined with the evidence that the avoidable admission of the palliative patient is a crucial point of the intervention [16,17], as it allows for its avoidance and better guidance of care.

In Portugal, we have seen significant growth in palliative care resources [18] with global coverage of hospital support palliative care teams and maintained deficits in community support. With an aging population and increased life expectancy, data from the 2023 Autumn Report of the Portuguese Observatory for Palliative Care shows that 11.2% of professionals working in PC and 10.3% of those working in Palliative Care Units still do not have specific training in PC for adults. The same study also states that global coverage of palliative care remains below what is desired, also revealing the focus on curative care, with insufficient specialized palliative care services in several regions, with a coverage rate of 9% for adults and only 0.9% for pediatrics [2,19].

The emergency care use in Portugal is particularly high (50 emergency department visits per 100 people), which could be seen as an indicative of inappropriate and inefficient healthcare, as was said in the OECD Report, Health at a Glance 2023, particularly if we are considering avoidable emergencies that can be managed in community care services [20].

The difficulties in managing existing resources are evident, with an increased aging population and with planning still poorly coordinated regarding the adequate structure for monitoring palliative needs. Investment in this area is urgent with strategic and articulated long-term support [19].

The aforementioned reinforces the study design by highlighting what is already a motivator for inclusion and better articulation between contexts, institutions, and palliative care teams: the reduction of economic costs.

The study starts with the central research question: What are the economic costs associated with nursing care provided to palliative patients who resort, in an avoidable emergency, to the general hospital emergency department?

The general objective is to determine the economic costs of nursing care provided to palliative patients upon admission to the general hospital emergency department in an avoidable emergency. To this end, the aim is to quantify the cost of nursing care provided to palliative patients, which is considered an avoidable emergency in a general hospital emergency department.

Knowledge of health costs will help to intervene with health decision-makers, highlighting the organization and quality of palliative care resources, namely, increasing teams specialized in palliative care.

## 2. Materials and Methods

The methodological aspects explored below were chosen to consider the study theme, the research question, and the proposed objectives.

This study is part of a thesis divided into three stages: the first, which starts from an integrative literature review (ILR) about the concept of “avoidable emergency” and continues to the second part, the Delphi process, used on the construction of the reference concept “avoidable emergency” [21] and the last one, the process of collecting and analyzing data related to cost economic benefits of nursing care for palliative patients in a hospital emergency department.

The study design was based on the contribution of national and international experts with whom the research project was shared, and the lines under development were discussed. From a micro perspective, the study collaborated with the research unit and hospital manager of the general emergency department of the hospital where the research took place, monitoring the data collection and processing process.

Therefore, without similar studies evaluating the cost of care in this context, our projection always considered the best theory on intervention in this context and research guidelines in palliative care.

A quantitative methodology was used, justified by the importance of specifying the data in numbers, essential elements in the description of the phenomenon under study, and the methodology of choice in most research studies in palliative care [22].

As for the data analysis, this is classified as a secondary study based on elements contained in a hospital database. Regarding the purpose, it is identified as an analytical study that compares hypotheses, verifies the existence of associations between variables, and evaluates nursing interventions. Moreover, in its directionality, longitudinal, as the data collected are recorded retrospectively, referring to the year 2019.

The study population was palliative patients who used the general emergency department of a hospital on the outskirts of one of the central metropolitan regions of mainland Portugal in 2019, with a total of 118.825 admissions to the general hospital emergency department. Considering the pandemic period from 2020 onwards, we ensured, through the selection of patients from 2019, the minor interference in the factors promoting avoidable admissions in general emergency department.

From the study population, a probabilistic sample was selected from patients who used the emergency department, considering the inclusion criteria: the population of patients from 2019, as this is the most recent year with data in the database complete and updated in the context of hospital admission and patients who required hospital emergency department and who ended up dying in that same service (“deceased–clinical autopsy”, “deceased–medico-legal autopsy” and “deceased without autopsy”), a choice that affected in the proximity of criteria capable of classifying patients as palliative and, therefore, eligible for the study.

In a sampling definition based on a population of 530 episodes/patients, the phenomenon formula of 55% and estimation error of 3% were applied, and a random set of 358 episodes was obtained for analysis. This sample was evaluated sequentially by a team of experts, presumed to be the hospital support palliative care team (HSPCT) from the same hospital from 11 May to 13 August 2020 and, subsequently, from 23 September to 30 December 2020. The evaluation of the HSPCT returned the identification of 274 palliative episodes/patients with potentially avoidable hospitalizations.

An avoidable emergency arises from any reason that can be supported in a home context or primary health care, especially when there is monitoring by a home palliative care team, as well as that which does not require immediate nursing and/or medical intervention nor does it translate into greater comfort or quality of life for the patient [21].

At each stage of the sample, the episodes relating to admission to emergency department were explored, one by one, with all the admission information, stay, and discharge being classified according to the type of patient and type of urgency; in each episode, the NECPAL 3.1 [23] tool was the resource chosen to verify the identification of the palliative patient, and after identifying as palliative, the admission was marked as “avoidable” or “not avoidable”, using the concept resulting from the ILR and the applied delphi process. All episodes could be identified as a case of doubt if there was any doubt regarding the classification of any of the previous parameters.

The sample of 274 episodes/patients was exhaustively evaluated in each process by the principal researcher, with the exclusion of one episode because it was an elective hospitalization, with direct entry to the inpatient service even if it was included in admissions to emergency department. The final sample, therefore, comprised 273 episodes/patients.

The data under analysis were made available by the hospital clinical informatization committee. The data processing intended to identify general aspects of admission to emergency department and characteristics of our sample, firstly, in demographic terms, and later, regarding the aspects characterizing admission—age, gender, origin, duration of complaints, the allocation, the clinical diagnosis group, the main reasons for admission and reason for discharge—and, finally, identify the nursing interventions carried out. Considering them, it was expected that there could be fixed values for each of the interventions identified.

We sought to validate whether there was already information regarding the costs of nursing care, using the hospital manager/nurse director of the institution where the study took place; researchers in the field of economics, namely from reference schools in public health, to nursing managers/responsible people of primary health institutions; to managers/nurse director of private hospital institutions and, finally, to the nurse director of a vital hospital center. The answer was always negative.

Given the absence of analytical accounting data regarding nursing care, the need to create them became apparent. In this way, three aspects were taken into account when considering the economic cost: the human factor—including the cost attributed to the nurse’s work—preparation/intervention, and registration; the material factor—resulting from the cost attributed to consumables used in nursing interventions and the structural factor—costs attributable to the institution and elements external to care, namely, direct fixed and variable costs.

In this way, the set of consumables necessary for its execution was structured for each identified nursing intervention based on the manual of standards, technical procedures of the Central Administration of the Health System, I.P. (ACSS) [24]. Data on consumable costs, as well as information on the value/hour of the emergency department nurse with social charges and direct costs, were made available by the Manager of the hospital where the study took place and by the nurse director of another hospital center, with a view to the complementarity of data, with the former being prioritized in the calculation of costs.

The application of descriptive statistics included elements of absolute and relative frequency (categorical variables) and the mean, median, standard deviation, quartiles, minimum, and maximum for the continuous variables. The distribution normality was analyzed (Kolmogorov–Smirnov test with Lilliefors correction) before choosing the best measure to characterize them. Facing the non-normality, the median and the interquartile range were the choices. Regarding inferential statistics, after analyzing the conditions to use or not use parametric tests, considering that the conditions were not present, we used non-parametric tests (Spearman correlations, Mann–Whitney test, and Kruskal–Wallis’s test) for the continuous and ordinal variables; for the nominal variables, the Qui-Square test was used. The statistical significance was defined by *p* < 0.05. All collected data were analyzed using Microsoft Office Excel^®^ and the Statistical Package for the Social Sciences^®^ version 25 (SPSS).

The aspects explored in SPSS included time and cost variables (Appendix A), identifying the “Emergency Time” (ET), which is understood as the length of stay of patients in emergency department, in minutes, from admission to discharge calculated by the difference between the discharge date and the admission date; “Intervention Time” (IT) which includes the time dedicated by the nurse to direct intervention with the patient, and the “Supervision Time” (ST) which considers the time dedicated monitoring/supervision of the patient.

In correspondence, we recorded the following cost variables: the “Cost of Intervention Time” (CIT), which corresponds to the cost of nursing care about the direct provision of care; the “Cost of Supervision Time” (CST), understood as the cost associated with the supervision of nursing care; the “Cost of Consumables” (CC) which is equivalent to the sum of the economic cost of all consumables per intervention listed; the “Direct Costs” (DC) understood as the costs relating to the emergency department’s fixed and semi-fixed cost values, data provided by the hospital manager, and the “Total Cost” (CT) encompassing the result of the sum of all previously presented costs.

## 3. Results

The sample considered 273 of 358 patients in the study (76.3%, CI 95%: 71.7–80.8), with one patient excluded due to elective hospitalization in an internal medicine service without staying in an emergency department.

We divided the study results into sample characterization elements, admission data, and cost definition data.

### 3.1. Sample Characterization and Admission Data

Of the 273 patients who made up the sample under study, their ages ranged between 37 and 102 years, with a median of 84 years. Regarding gender, it was found that 50.2% of patients were female (n = 137) and 49.8% male (n = 136). Regarding the origin, there were statistically significant differences (x^2^(2) = 232.286, *p* < 0.001) in the place of origin of the patients admitted to hospital emergency, with a percentage of 75.5% (n = 206) of the patients coming from home, from nursing homes 21.6% (n = 59), and the hospital in 2.9% (n = 8) of the episodes.

Of the patients in the sample (n = 273), all were evaluated in screening; however, 49.1% (n = 134) were observed in the resuscitation room. There was no statistically significant difference between the group assisted in resuscitation and the group not assisted in resuscitation (*p* = 0.809). Regarding the duration of patients’ complaints (hours) justifying hospital admission, the median was 5 h, with a minimum of 0 and a maximum of 168 h. Of the patients evaluated, 96.3% (n = 263) were admitted to the general observation service, while 3.7% (n = 10) remained at the general emergency desk.

About the clinical diagnosis groups of patients, statistically significant differences were recorded (x^2^(11) = 1819.703, *p* < 0.001) with patients with diagnoses in the “others” group in a percentage of 79.5% (n = 217), malignant neoplasms (except leukemia) with 5.9% (n = 16), and lung diseases with 4.8% (n = 13). For the remaining clinical diagnoses, percentages ranged from 0.7% to 1.8% (n from 2 to 5) (Table 1).

In the reasons for admission of patients, the predominance of reference to respiratory symptoms was verified by 66.7% (n = 182), general malaise by 26.0% (n = 71), and others by 24.5% (n = 67). Of the signs/symptoms identified, the three main ones were considered, and the same patient may have had more than one reason for admission. The remaining signs/symptoms mentioned on admission varied between 13.2% in neurological symptoms and 0.7% in wounds (Table 2).

Inferential statistics were applied to the variables of time and costs compared with symptoms upon admission using the non-parametric Mann–Whitney test to identify the absence of normality in the distributions.

The data obtained indicated that respiratory symptoms generated statistically significant differences in the parameters: Time in emergency (U = 6740.000, *p* = 0.012|Mean Rank = 128.53 (With Symptom = Yes); Mean Rank = 153.93 (No Symptom = No)), supervision time (U = 6723.500, *p* = 0.011|Mean Rank = 128.44 (Yes); Mean Rank = 154.12 (No)), cost of supervision time (U = 6723.500, *p* = 0.011|Mean Rank = 128.44 (Yes); Mean Rank = 154.12 (No)), direct costs (U = 6740.000, *p* = 0.012|Mean Rank = 128.53 (With Symptom = Yes); Mean Rank = 153.93 (Without Symptom = No)) and total cost (U = 6782.000, *p* = 0.015|Mean Rank = 128.76 (Yes); Mean Rank = 153.47 (No)), with lower time values and costs for symptomatic patients.

Regarding the reason for admission—hemorrhage, statistically significant differences were generated about the variable intervention time (U = 1008.000, *p* = 0.014|Mean Rank = 84.54 (Yes); Mean Rank = 139.62 (No)), cost of intervention time ((U = 1008.000, *p* = 0.014|Mean Rank = 84.54 (Yes); Mean Rank = 139.62 (No)) and cost of consumables ((U = 1103.500, *p* = 0.035|Mean Rank = 91.88 (Yes); Mean Rank = 139.26 (No)), with higher values being perceived in patients without evidence of hemorrhage. Regarding the reason for admission—general malaise, a statistically significant difference was evaluated in intervention time (U = 5638.500, *p* = 0.007|Mean Rank = 158.58 (Yes); Mean Rank = 129.41 (No)) and cost of intervention time, with the record of an increase in the time and cost of intervention in patients with this reference upon admission. Regarding gastrointestinal, neurological, urinary symptoms, pain, wounds, and others, there was no statistically significant difference in the relationship with the variables time and costs (*p* > 0.05).

The reason for discharge was for 99.6% of patients (n = 272) “Deceased without autopsy”, with 0.4% (n = 1) corresponding to “Deceased–Medico-legal autopsy”.

### 3.2. Cost Definiton Data

Regarding cost definition data, it is essential to note that time and cost variables were considered (Table 3).

When accounting for the costs of nursing care for palliative patients in the ES, we were able to identify the following:

- The “emergency time”: Total length of stay in emergency department since admission—a median of 1446 min, with a minimum of 0 min and a maximum of 17,082.60 min recorded;
- The “intervention time”: Total time, in minutes, of the set of nursing interventions carried out per patient—a median of 96 min was identified, with a minimum of 27 and a maximum of 492 min recorded;
- The “supervision time” corresponding to the difference between “urgency time” (minutes) and “intervention time”—a median of 1363 min was identified, with a minimum of 0 min and a maximum of 16,943.60 min registered;
- As for the “cost of intervention time”, it was obtained from the product of the “intervention time” by the factor 0.17 (EUR/min) (previously defined as the cost of work per minute)—the median recorded was EUR 16.32, with a minimum of EUR 4.59 and a maximum of EUR 83.64;
- The “cost of supervision time” was obtained from the product of “supervision time” by the factor 0.053 (EUR/min) (previously defined as the cost of supervision per minute)—had a median of EUR 72.24, a minimum of EUR 0 and a maximum of EUR 898.01 counted;
- The “cost of consumables” was the sum of all consumables used in nursing interventions—a median of EUR 35.97 was obtained, a minimum of EUR 9.77, and a maximum of EUR 157.66 registered;
- The “direct costs” were understood as costs relating to variable and semi-fixed values, subsequently considered in the product of “urgency time” for 0.036 (EUR/min), previously obtained as the value of direct costs per minute evaluated with a median of EUR 52.06, a minimum of EUR 0, and a maximum of EUR 614.974;
- The “total cost” comprising the sum of all previously presented costs was a median of EUR 180.98, a minimum of EUR 14.36, and a maximum of EUR 1588.604.

Considering the data presented above, correlations between quantitative variables were explored using the Spearman correlation. This coefficient was used to evaluate the intensity of the relationship between non-normal and ordinal variables (Table 4).

There are moderate positive correlations between urgency time and intervention time, cost of intervention time and consumables, and robust positive correlations between emergency time and supervision time, cost of supervision time, direct costs, and total cost.

Regarding intervention time, there were moderate positive correlations with all time and cost variables, except for the variables cost of intervention time with a robust positive correlation and the costs of consumables in which an assessment is made. Strong correlation: as far as supervision time is concerned, the opposite of what was described above occurs, with robust positive correlations with all variables except for intervention time, cost of intervention time, and consumables involved. We evaluated moderate positive correlations.

Considering the cost of intervention time, moderate positive correlations were evaluated with the cost of supervision time, direct costs, and total cost, with the correlation with consumable costs being a strong positive correlation. Regarding the cost of supervision time, the correlation is positive and moderate with the costs of consumables and cheerful and very strong with the variable’s direct costs and total cost. A moderate positive correlation is identified between direct and total costs when assessing consumable costs. Direct costs and total costs have a robust positive correlation. Concerning the duration of complaints (CDs), weak positive correlations were found with all other variables (Table 4).

The comparison between the time and cost variables and the patient’s origin (non-normal variables) started by applying the Kruskal–Wallis non-parametric test, and it was possible to conclude that there were no statistically significant differences between them.

The sample of the hospital’s origin (n = 8) was tiny, preventing us from concluding. Therefore, a new Kruskal–Wallis test was used to verify the comparison between the time and cost variables and the patient’s origin limited to the house or nursing home, concluding that there were no statistically significant differences.

Regarding the comparison between the variables, duration of complaints, and origin, applying the non-parametric Kruskal–Wallis test, given that the distributions evaluated did not present normality, it was concluded that there are statistically significant differences between these (x^2^kw(2) = 24.556, *p* = 0.000, *p* < 0.05).

We were able to determine that the duration of complaints was higher when originating at home (Mean Rank = 144.91), shorter when originating at the nursing home (Mean Rank = 126.58), and even lower if the patients came from other hospitals (Mean Rank = 10.19).

## 4. Discussion

In contextualizing the data analysis, framing a set of study limitations will be relevant. Regarding the sample type, it was a limitation in the random sample that patients who died in 2019 in the emergency department were included. This option limited data analysis to the files of patients in their last months of life whose reason for discharge was death. This option was the one that best approached the sample of patients with palliative needs, as there is no coding for palliative patients [25] in health services, and it would be unfeasible to apply the NECPAL 3.1 tool [23] to the entire population admitted to the emergency department in 2019. This retrospective consideration reduced the risk of bias between the number of episodes and possible repeat patients.

Another initial limitation was the use of only one hospital for this study. Considering the difficulties in achieving precise hospital data and the limited research team integrated into this thesis study, this was the best approach for the first study type known, like this one in Portugal.

Also, a single palliative care team was used, in this case, the HSPCT of the hospital institution where the study occurred. These were the hospital’s only specialized palliative care resources. This limitation was understood as a positive point, considering data availability by a group of professionals who remained stable during the different stages of analysis, overcoming any bias that the change of professionals and perspectives could cause.

Still, in the data analysis, considering the lack of more precise information in defining the “avoidable urgency” concept, we constructed it after an ILR and a delphi process. Without a standard definition, the one created may or may not have caused an undue consideration of the number of cases eligible as preventable. In any case, as we will see later in the data analysis, the percentages obtained are part of the consideration of international literature [26]. We are not aware of the existence of this information in a national context.

Considering the data evaluation, limitations are assumed, such as the regrouping of less prevalent complaints in the “others” group regarding reasons for admission and limitations in cost accounting, given the lack of organized, analytical accounting in nursing care. The possible overestimation of intervention times may also be a bias in our study because in estimating supervision times, there was a need in cases of patients with little or practically no time in the hospital (imminent terminal situations admitted to the hospital, resuscitation, for example), assuming them as zero, when the actual value would be negative but unfeasible to count (if the emergency time were shorter than the intervention time, the supervision time assumed would be zero).

References to the most recent direct costs, namely medication, exams, or transport, were not related to the same year of the study, 2019, a limitation circumvented by including inflation quotes. The lack of information on water or electricity costs, medication costs, or costs associated with using a laboratory or imaging was also not included due to the difficulty of accessing this information.

### 4.1. Sample Characterization

The 273 patients in the study sample had a median age of 84 years. The problem of aging as a characteristic of a considerable increase in patients in emergency departments is a growing and increasingly common phenomenon [27,28]. In the USA, half of people aged 65 or over attend a hospital emergency room in the last month of life, and in Great Britain, the phenomenon is on the rise when analyzing the last year of life [28]. Considering that our sample corresponds to hospital deaths. It is essential to recognize the future perspectives that indicate an increase of more than a quarter in hospital deaths by 2030 [29].

Regarding gender, it was noticed that there were no differences between both proportions, and the homogeneity of the sample can be affirmed. Even so, according to the evidence, there may be a prevalence of male admissions in emergencies by palliative patients [13,30].

### 4.2. Admission Details

The admission of patients under study began with screening, of which almost half were directed to the resuscitation room.

Upon hospital admission, we understood that 134 patients identified as palliative received immediate support measures in the resuscitation room. According to the statistical evaluation, no statistically significant differences were assessed between groups regarding whether or not they were assisted in the resuscitation room. Regarding this specific aspect, no relevant data were found in the literature. However, based on the patients’ lifetime monitoring, these measures may have constituted dysthanasic interventions or aggressive care [15,31].

Regarding the place of origin, there were statistically significant differences, realizing that 3/4 of the patients were admitted from home, 1/5 from the nursing home, and almost 3% from the hospital, with the latter resulting from hospital transfers, in many cases, because it is the hospital in the area of residence; these data were in line with the evidence [32,33].

Home, often discussed in the transition to the emergency department, is also the preferred care space. Most of the patients desire to die at home [27,29,34,35,36,37,38]. In Portugal, this option represents around 51.2% [39] and, according to the evidence, with no change in consideration as the disease progresses [36]. It is recognized, in the evidence, that patients monitored by palliative care teams are more likely to die at home if that is their wish [13,40,41].

Understanding that all the episodes under study are characterized as avoidable admissions, the duration of patients’ complaints, in hours, was estimated at a median of 5 h. We can assume that patients presented a long period of complaints before resorting to ED, which may lead us to question why they did not use other means of assistance or whether they were available.

The evidence indicates that one of the aspects that promote admission to the emergency department is mainly due to the absence of home care and palliative care [35,38,42], namely, due to lack of support or incomplete monitoring period, considering the 24 h and 365 days of the year [4,43]. Another aspect that promotes these transfers to the hospital is the “pressure” experienced by health professionals contacted during acute situations who are unaware of the defined care plan [13]. Still, family members’ desires can be decisive in transitions to the hospital space, such as social isolation or the absence of a caregiver [44,45].

The importance of integrating palliative care in the emergency departments and a hospital context can be seen by verifying all the benefits of their intervention and support for crisis episodes, from which the transition takes place [16,46]; however, in emergency departments, palliative knowledge is limited, with an intervention and healing culture, health professionals with little or no training in palliative care and prevalence of medico-legal concerns [17,47,48].

There are inevitable and justified admissions or hospitalizations. However, the evidence shows that palliative care patients experience significantly fewer adverse effects throughout their illness, have a higher quality of life, and are less likely to avoidable admissions, unnecessary diagnoses, therapeutic procedures, hospitalizations, and hospital deaths [3,4,5].

Timely referral to palliative care guarantees the possibility of intervening in complex needs, promoting relief from suffering and comfort for patients and families. In this sense, costs are reduced by reducing so-called futile interventions and hospitalization times, collaborating with patients’ preferences and needs. This significantly impacts the patient’s health journey [49,50,51,52,53].

The contrary trend to this phenomenon is defended through community palliative care teams, for which the evidence shows lower numbers of hospital emergency admissions and an equal impact on reducing lengths of stay [4,54].

Upon their hospital admission, the patients in our sample were integrated into the emergency department in two locations: the emergency desks, around 4%—outpatient area—and the observation service, more than 95%—inpatient area.

Concerning the clinical aspects characterizing the health process of these patients, we were able to conclude that there were statistically significant differences between patients with diagnoses in the “other” group of 80%, malignant neoplasms (except leukemia) with 6%, and lung diseases with 5%. With these three main diagnostic groups defined, the remaining clinical diagnoses revealed percentages of two to five patients in the sample.

According to administrative information, the largest group, “others”, includes a set of non-concrete diagnoses. Thus, we were able to infer about the lung diseases and malignant neoplasms identified, which both appear in the primary diagnostic groups for admission of patients to emergency departments, so it can be concluded that our results follow the scientific trends already studied [13,34,45,55,56].

Regarding the reasons for admission, results similar to those in the evidence were identified [37,57], with a predominance of complaints due to respiratory symptoms at 70%, general malaise at around 30% of the sample, and others at practically 25%. It should be noted that several signs/symptoms were attributed to each process, as identified in screening, and a selection was decided into the main groups of three. Still, regarding the process of defining the groups, they resulted from the grouping of complaints such as “dyspnea”, “respiratory difficulty”, “polypnea”, and “cough” in respiratory symptoms; “prostration”, “indisposition”, “asthenia” in general malaise; “fall”, “decrease in limb strength”, “food refusal”, “fever”, “therapy refusal”, “choking”, “tachycardia”, “edema”, “hyperglycemia”, “Implantable cardioverter-defibrillator”, “jaundice”, “dehydration”, “pruritus”, “food aspiration” and/or “palpitations” in others.

The other highlighted signs and symptoms varied in percentages, around 10% in neurological symptoms and less than 10% in wounds.

These admissions due to lack of symptomatic control are one of the main motivations for hospital admission [44,55,58], with pain being one of the constant complaints (in our sample with a representation of 1/10) [57], but dyspnea or difficulty breathing proved to be prevalent in the motivation for going to a hospital emergency [37].

From the signs and symptoms identified, the comparison between these and this study’s time and cost parameters was evaluated using inferential statistics.

The results indicated that respiratory symptoms generated statistical differences in the patient’s length of stay in the emergency room, the time allocated to nursing supervision, the cost of supervision time, direct costs, and total costs. It was identified that patients with respiratory symptoms have associated lower costs and times. This may be associated with symptoms in the last days/hours of life due to a group of patients with a predominance of respiratory complaints and consistent diagnoses and, therefore, less effective time in emergency care, with reduced interventions and associated costs. Indeed, lung diseases in terminally ill patients tend to be quite aggressive, with high mortality rates [59].

The reason for admission, hemorrhage, generated statistically significant differences in the parameters of time allocated to nursing interventions per patient, in the cost allocated to intervention time per patient, and in the total cost of consumables; intervention time and costs were lower in patients without signs of bleeding, except for the costs of consumables, the latter being higher in patients with bleeding complaints. Considering intervention in palliative urgency, it is understood that the response to a massive hemorrhage will imply the replacement of fluids and care to contain the visual presentation of the same and hemostatic containment [60,61,62].

No statistically significant differences were evaluated regarding time and costs of gastrointestinal, neurological, urinary symptoms, pain, wounds, and others (*p* > 0.05).

All patients in our sample were discharged after death, verifying that only one patient corresponded to the “Deceased–Medico-legal Autopsy” classification, with the rest being discharged as “Deceased without autopsy”.

### 4.3. Cost Definition Data

Regarding cost definition data, the variables time and costs were considered.

When accounting for nursing care costs for palliative patients, we identified the total length of stay in emergency department, a median of 1446 min or 24.10 h. This period indicates that many of the admissions associated with palliative patients occurred close to the end of life, with death occurring within a day, as identified in the literature, with 40% of patients attending a hospital emergency room in their last two weeks of life or even in their last days of life [57,63]. In a study with 48 patients, an average of 596 min was spent in the emergency department, with 50% of this sample remaining between 120 and 330 min; only two patients died in the emergency department [45].

During the time in the emergency department, supervision time was distinguished from intervention time by the nurse. The first is estimated at 1363.00 min or 22.72 h, that is, time without direct intervention but time spent monitoring and recording care. The intervention time was estimated at 96 min or 1.60 h, including the exercise of a set of interventions previously identified as referenced in the methodology.

Regarding costs, in the form of a median, we found that the cost of intervention time was EUR 16.32, with the cost of supervision time estimated at EUR 72.24. Regarding the costs of consumables, the value was EUR 35.97. Direct costs are considered EUR 52.06. Therefore, the total cost comprised the sum of all previously presented costs and was estimated at a median of EUR 180.98 and an average of EUR 251.94 per episode of admission of a palliative patient in an avoidable emergency.

Still, inferential statistics were applied in the above data, using Spearman correlation to assess the intensity of the relationship between non-normal and ordinal variables of time and costs. It was considered that there was a robust, statistically significant correlation between the variables considering *p* ≤ 0.01. We concluded that the longer the stay or intervention/supervision, the higher the costs of intervention/supervision times and direct and total costs.

Regarding times and costs, these demonstrated a statistically significant correlation with the duration of complaints, verifying that the longer the duration of complaints, the greater the time and cost associated with hospital admission. Based on these conclusions, it was considered logical that a length of stay, intervention, or supervision would incur more significant costs in all aspects studied, knowing that the emergency department is a space that privileges the response to disease problems acute and poorly structured to respond to palliative needs [59,61].

As for the duration of complaints being proportional to the time and costs, we can infer that a longer waiting time for resolving complaints in patients with palliative needs will correspond to a more complex problem and with a greater need for intervention so that the time and associated costs will be significantly greater. We are unaware of a study that has obtained results on this type of inference.

Regarding comparing the variables, duration of complaints, and origin, statistically significant differences were verified using the Kruskal–Wallis test. It was assessed that the duration of complaints was higher at home, shorter when originating at a nursing home, and even shorter if the patients came from other hospitals, the latter sample being tiny. These results align with the consideration that the more specialized the places of origin, the shorter the duration of complaints, assuming the existence of a supportive response to problems, in this case, in other hospitals, in nursing homes, and, finally, at home. A study identifies nursing homes as an element of predisposition to an avoidable admission. However, we did not find data regarding home origin [64].

According to some authors [65], health costs increase in the last 12 months of life and can be four times higher in the last 6 months. Despite being a poorly understood phenomenon [65], it is recognized that the cost of hospital death is twice as high as death at home [66].

The study by The Lancet Commission on the Value of Death recognizes a higher health cost in the last 30 days of patients’ lives-in six developed countries-this period being disproportionately higher compared to the estimate for the last 6 months of life. The average cost of expenses in the last 30 days of life of patients who died with oncological disease was estimated at USD 4766 (The Netherlands), with a percentage of hospital death of 24.6% among patients with any disease with palliative needs; USD 6206 with 50.8% hospital death (Belgium); USD 10,843 with 61.4% hospital death (Canada), and USD 6934 associated with 47.5% hospital death (England) [67].

The study by Penrod et al. is the only one that, to our knowledge, distinguishes the costs of nursing care for patients with an advanced illness, and its objective was to indicate the value of savings between palliative care and usual care. There were savings results, in dollars, in the order of USD 182 (CI95%: USD 201–164), which converted to EUR at the current rate, corresponding to EUR 168 (CI95%: EUR 185–151). How care costs are estimated is unknown, as are the usual values [68].

As for the economic impact of reducing avoidable emergencies, this was studied in Great Britain [35], concluding that increasing community resources could save costs associated with avoidable hospital admissions. The costs incurred with emergencies for palliative patients in the last year of life would total around GBP 1.3 billion. In this study, the percentage of avoidable admissions was 6.7% (14 patients out of 580 in the sample) with an average of GBP 2595 (ranging from GBP 451 to 5363), considering only emergencies that could not be answered elsewhere other than hospital ones. Preventing these admissions and supporting these patients in the community would correspond to saving for both study hospitals GBP 1527 over the study period or GBP 180.000/year.

A retrospective observational study covering two hospitals in the north of England highlights in its sample of 483 patients who died within a year of hospital admission that avoiding these admissions and the possibility of being cared for in other spaces would allow savings of GBP 5.9 million per year. Reducing hospital stays by 14% would result in around GBP 47.5 million annual savings. This would not consider possible increases in the costs of community care, considering, from now on, the accuracy of home care data as a limitation [12].

Another retrospective study [26] meets our results; it analyzed data from 4346 patients of a Korean tertiary hospital, identifying 55.7% of avoidable admissions. The average cost per avoidable admission was concentrated at USD 369.80 and annually at USD 894.877.

A comparative analysis between the costs of usual care and the costs of care with intervention by a palliative care consultant team identified a total cost per day in usual care of USD 2468 (2332–2603) and savings in the intervention of the palliative care team [69].

Cost and efficiency assessment studies could have been more representative due to small samples, methodological flaws highlighted, and little diversity [70]. The data obtained regarding end-of-life costs proved to be controversial and, at the same time, a little-understood phenomenon [65]. The evidence is scarce [71,72,73], and the variation in costs is significant, with few studies considering the cost of care provided by informal caregivers or other cost components, such as nursing care [74].

Moreover, the evidence highlights the heterogeneity between publications, increasing difficulties in data interconnection. In that case, another study explores the increase in studies on economics in palliative care in the last ten years [25,74], indicating a savings pattern but with diverse factors. The same authors identify savings as positive and the results as at least as good as expected. Even so, these are the results of costs tending to be limited to a short period, omitting costs per user and family, with centralization, as in our study, in accounting for short death processes.

Some authors, however, noted savings due to admission and considered accounting for savings through consultation with the team [68]. Regarding this, they report that the cost reduction is associated with reduced requests to the laboratory, complementary exams [53], intensive care, direct costs per admission (which our study explores), and daily costs. They also conclude with more significant savings in patients who died in hospital, as in our sample, focusing on the positive influence that palliative support revealed regarding futile intervention in palliative patients and during hospitalization times [47,69].

Still in the process of studying the economic impact on palliative care, some difficulties stand out, such as analysis due to the lack of coding in palliative care, as we mentioned in limitations, difficulties due to different contexts, the diversity of interventions, the analysis of patients, and methodological difficulties [25].

The importance of identifying all cost components used in hospital palliative care is highlighted, and of all the studies read, only one includes the costs of nursing care in the care of palliative patients, referring to it as being one of the most significant cost components in supporting these patients [33].

The social and financial pressure on health systems signals a growing trend, namely due to the future challenges already mentioned, making it vital to understand how to invest the best resources that are currently limited to achieve the best results in palliative care for patients, families, caregivers, clinicians, and political decision-makers [12,75]. It is important to remember that no direct scientifically consolidated relationship exists between more costs/expenses and better care [76].

## 5. Conclusions

The cost of nursing care is a new perspective that this study explores concerning the palliative care field. The cost of palliative patients undergoing avoidable admission in the emergency warrants its relevance in the health organization of services and palliative care integration into the health system.

Future studies should consider concrete data in health contexts and privileged longitudinal studies in the nursing economics of palliative care.

With a sample of 273 palliative patients in preventable hospital admissions (76.3%, CI 95%: 71.7–80.8), this study shows that the significant emergencies are mainly due to respiratory symptoms, resulting in high nursing costs. A median of EUR 180.98 in nursing care costs per admission was determined, reinforcing the need for better coordination between institutions and contexts to reduce costs and improve end-of-life care.

This finding ensures the attention of palliative care representatives and health policymakers to create future palliative care policies.

## Figures and Tables

**Table 1:** Clinical group diagnosis.

Measure	Value	%
Malignant neoplasms (except leukemia)	16	5.9
Lung diseases	13	4.8
Kidney failure	5	1.8
Non-ischemic heart disease	5	1.8
Arteriosclerosis	4	1.5
Hemorrhagic Fever	3	1.1
Dementia	2	0.7
Chronic ischemic heart disease	2	0.7
Cerebrovascular diseases	2	0.7
CNS Degenerative diseases	2	0.7
Liver diseases	2	0.7
Others	217	79.5
Total	273	100

**Table 2:** Reasons for patient’s admissions.

Measure	Value	%
Respiratory symptoms	182	66.7
General malaise	71	26.0
Others	67	24.5
Neurological symptoms	36	13.2
Gastrointestinal symptoms	33	12.1
Pain	33	12.1
Bleeding	13	4.8
Urinary symptoms	7	2.6
Wounds	2	0.7

**Table 3:** Cost definitions.

Costs Definitions	Median	Minimum	Maximum
Emergency time (min)	1446	0	17,082.60
Intervention time (min)	96	27	492
Supervision time (min)	1363	0	16,943.60
Cost of intervention time (EUR)	16.32	4.59	83.64
Cost of supervision time (EUR)	72.24	0	898.01
Cost of consumables (EUR)	35.97	9.77	157.66
Direct costs (EUR)	52.06	0	614.974
Total cost (EUR)	180.98	14.36	1588.604

**Table 4:** Spearman correlation coefficients.

	ET	IT	ST	CIT	CST	CC	CD	CT	Age	CDs
Emergency time (ET)		0.630 *	0.999 *	0.630 *	0.999 *	0.509 *	1.000 *	0.993 *		0.297 *
Intervention time (IT)			0.613 *	1.000 *	0.613 *	0.854 *	0.630 *	0.689 *		0.274 *
Supervision time (ST)				0.613 *	1.000 *	0.494 *	0.999 *	0.989 *		0.293 *
Cost of intervention time (CIT)					0.613 *	0.854 *	0.630 *	0.689 *		0.274 *
Cost of supervision time (CST)						0.494 *	0.999 *	0.989 *		0.293 *
Cost of consumables (CC)							0.509 *	0.583 *		0.251 *
Direct costs (CD)								0.993 *		0.297 *
Total cost (TC)										0.302 *

* Significant correlation for *p* ≤ 0.01.

## Data Availability

The research data in this study are available on request from the corresponding author.

## Appendix A. Time and Costs Explanations

The aspects explored in SPSS included time and cost variables, identifying the following:

In the accounting process, considering the cost per consumables and gathering the consumables per intervention, we obtained the cost per intervention; however, a relevant aspect was missing in the definition of economic cost—the time factor.

Therefore, the participation of the emergency department chief nurse of the hospital where the study took place was requested, as well as the responsible nurses, team leaders, and other nurses on the team. From these, the total number of emergency department nurses working in 2019 was determined as 142, the average number of nurses per team was 58, and, finally, data on the time considered for the preparation/execution and recording of each intervention, obtained by averaging a set of responses from 13 nurses, in an online form using Google Forms.

Other time and cost variables were as follows:

- “Emergency Time” (ET), which is understood as the length of stay of patients in emergency department, in minutes, from admission to discharge, calculated by the difference between the discharge date and the admission date;
- “Intervention Time” (IT), which includes the time dedicated by the nurse to direct intervention with the patient, namely in the different procedures previously listed—calculated through the average of the definition of intervention time defined by the emergency department nurses;
- Finally, the “Supervision Time” (ST), which considers the remaining period of the nurse’s intervention with the patient in minutes, in addition to the time due to the intervention in the procedure; therefore, the time dedicated to monitoring/supervision of the patient—calculated by the difference between the “Emergency Time” and the “Intervention Time”.

In cases of negative estimated supervision times (if ET < IT, ST = 0), it was decided to consider it as zero (cases of patients admitted in cardio-respiratory arrest or patients who died shortly, the time for standard interventions exceeded the length of stay in the emergency room).

In correspondence, we recorded the following cost variables:

- The “Cost of Intervention Time” (CIT), which corresponds to the cost of nursing care about the direct provision of care and, thus, the result of the product of “Intervention Time” by the factor 0.17 (EUR/minutes). This last value is calculated as the cost of work per minute, considering the given data of EUR 9.92 in the nurse’s hourly rate already with social charges (EUR 9.92/60min = EUR 0.17/min);
- The “Cost of Supervision Time” (CST) is understood as the cost associated with the supervision of nursing care, calculated by the product of the “Supervision Time” and the factor of EUR 0.053. This value was derived from the division of the average cost per patient hour in the ED, EUR 3.19 [value/hour of the emergency department nurse * total hours of weekly working hours * total working weeks/year * total number of ED nurses in 2019/total number of hours of patients admitted to emergency department in 2019/60 min—9.92*36*52*142/826949.10/60 = 0.053 EUR/min.];
- The “Cost of Consumables” (CC), which is equivalent to the sum of the economic cost of all consumables per intervention listed;
- “Direct Costs” (DC) are understood as costs relating to the emergency department’s fixed and semi-fixed cost values, which are data provided by the hospital manager. These costs per patient minute in emergency department were subsequently considered. The calculation of this value resulted from the product of “Emergency Time” by 0.036 (EUR/min), a value previously obtained as the value of direct costs per minute from the division of the sum of variable direct costs and semi-fixed direct costs (the most recent data EUR 1,669,948 + EUR 75,645), a value to which the inflation was added, totaling EUR 1,768,338, with the total number of minutes of the total set of patients admitted to emergency department in 2019 (49,571,014.2 min). These costs included variable direct costs—medicines, clinical consumables, reagents, other material, external exams, user transport, food, laundry, and cleaning—and semi-fixed direct costs—communication, surveillance and security, conservation and repair, specialized work, and other supplies and services. The variable costs did not identify the value of goods, blood, and hospitalizations abroad. In the semi-fixed costs, the value of electricity, fuel, water, and insurance was not identified;
- The “Total Cost” (TC) encompasses the result of the sum of all previously presented costs.
